# Effects of combined dietary intervention and physical-cognitive exercise on cognitive function and cardiometabolic health of postmenopausal women with obesity: a randomized controlled trial

**DOI:** 10.1186/s12966-024-01580-z

**Published:** 2024-03-05

**Authors:** Puntarik Keawtep, Somporn Sungkarat, Sirinun Boripuntakul, Piangkwan Sa-nguanmoo, Wanachaporn Wichayanrat, Siriporn C. Chattipakorn, Puangsoi Worakul

**Affiliations:** 1 Department of Physical Therapy, Faculty of Associated Medical Sciences, Chiang Mai University, Chiang Mai, Thailand; 2 A Research Group of Modern Management and Information Technology, College of Arts, Media and Technology, Chiang Mai University, Chiang Mai, Thailand; 3 Neurophysiology Unit, Cardiac Electrophysiology Research and Training Center, Faculty of Medicine, Chiang Mai University, Chiang Mai, Thailand; 4 Department of Oral Biology and Diagnostic Sciences, Faculty of Dentistry, Chiang Mai University, Chiang Mai, Thailand; 5 Clinical Psychology Program, Faculty of Education, Prince of Songkla University, Pattani Campus, Songkhla, Thailand

**Keywords:** Cognitive function, Physical-cognitive, Exercise, Diet, Intermittent fasting, Executive function, Adiponectin, Postmenopausal women, Obesity

## Abstract

**Background:**

Postmenopausal women with obesity are markedly at risk of cognitive impairment and several health issues. Emerging evidence demonstrated that both diet and exercise, particularly physical-cognitive exercise are involved in cognitive and health benefits. However, the comparative effect of diet, exercise, and combined interventions in postmenopausal women with obesity on cognition and cardiometabolic health is still lacking. Identifying the effective health promotion program and understanding changes in cardiometabolic health linking these interventions to cognition would have important medical implications. This RCT aimed to examine the effect of single and combined interventions of diet and exercise on cognitive function and cardiometabolic health in postmenopausal women with obesity.

**Methods:**

Ninety-two postmenopausal women with obesity were randomly assigned to diet group (intermittent fasting 2 days/week, 3 months), exercise group (physical-cognitive exercise 3 days/week, 3 months), combined group, or control group (*n* = 23/group). All cognitive outcomes and cardiometabolic outcomes were measured at baseline and post-3 months. Primary outcomes were executive functions, memory, and plasma BDNF levels. Secondary outcomes were global cognition, attention, language domain, plasma adiponectin levels, IL-6 levels, metabolic parameters, and physical function.

**Results:**

At the end of the 3-month intervention, the exercise and combined group demonstrated significant memory improvement which was accompanied by significant improvements in plasma BDNF level, insulin levels, HOMA-IR, %body fat, and muscle strength when compared to controls (*p* < 0.05). Only the combined intervention group demonstrated a significant improvement in executive function and increased plasma adiponectin levels when compared to control (*p* < 0.05). Surprisingly, no cognitive improvement was observed in the diet group (*p* > 0.05). Significant reduction in cholesterol levels was shown in the diet and combined groups when compared to controls (*p* < 0.05). Among the three intervention groups, there were no significant differences in all cognitive outcomes and cardiometabolic outcomes (*p* > 0.05). However, all three intervention groups showed significant improvements in plasma BDNF levels, weight, BMI, WHR, fat mass, and predicted VO_2_ max, when compared to control (*p* < 0.05).

**Conclusion:**

These findings suggest that combined physical-cognitive exercise and dietary intervention are promising interventions to improve cognition and obesity-related complications of postmenopausal women with obesity.

**Trial registration:**

NCT04768725 (https://clinicaltrials.gov) 24th February 2021.

**Supplementary Information:**

The online version contains supplementary material available at 10.1186/s12966-024-01580-z.

## Introduction

Globally, the prevalence of obesity in women has dramatically escalated in all ages and ethnicities [1]. Obesity has negative impacts not only on physical and cardiometabolic health but also on cognitive performance [2]. Accumulating evidence has revealed that obesity is linked to deficits in the global cognition [3] and multiple cognitive domains (e.g., executive function [3, 4], memory [5], and language domain [5]). While the mechanisms by which obese-induced cognitive decline are not well elucidated, pathophysiological changes including insulin resistance, adiponectin dysregulation, and systemic inflammation have been suggested as key mediators in the reduction of neurotrophic factors, neurogenesis, and consequently lead to cognitive impairment in obesity [3, 4, 6]. Besides the obese condition, the influence of estrogen deprivation is also associated with a reduction in cognitive performance [7]. As estrogen hormone has profound effects on neuroplasticity via the synthesis of neurotrophic factors and synaptogenesis, estrogen deprivation due to menopause could lead to cognitive impairment [7, 8]. Thus, postmenopausal women with obesity are at increased risk for cognitive impairment and health problems.

Recommendations to manage obesity are reducing caloric intake and enhancing physical activity. Intermittent fasting (IF) is one of the popular dietary interventions that promote dietary compliance, weight loss, metabolic, and health benefits [9, 10]. Moreover, emerging evidence has further demonstrated the benefits of IF on cognitive function in adults [11], and older adults with and without health conditions [12]. Nevertheless, some studies did not find cognitive improvement after IF intervention [13, 14]. The discrepancy in previous findings could be attributed to differences in the cognitive assessment, regimen of IF, and research design. Currently, clinical trials examining the effects of dietary intervention with IF method on cognitive function in postmenopausal women with obesity are limited.

Physical exercise is an effective strategy to enhance cardiovascular fitness, reduce obese-related complications, as well as improve cognitive function [15]. Among several exercise strategies that focus on cognitive benefits, a growing body of evidence has demonstrated that physical-cognitive exercise (dual-task training e.g., animal naming while performing step exercise or sequential motor-cognitive training e.g., bicycling followed by solving proverbs with missing words and letters) provides better cognitive benefits than physical exercise alone [16, 17]. The positive effects of physical-cognitive exercise on global cognition and specific cognitive domains including executive function and memory have been reported in all age groups, especially in older adults with and without cognitive impairment [18–20]. Few studies have investigated the effects of physical-cognitive exercise on cognition in obesity with postmenopausal condition [21]. In addition, evidence has suggested that both exercise and cognitive training are involved in the improvement of cardiometabolic health and neural mechanisms (e.g., the improvement of neurotrophic factors and neurogenesis), and may consequently lead to cognitive improvement [22–24].

Taken together, this study aimed to examine the effects of dietary intervention, physical-cognitive exercise, and combined intervention on cognitive function of postmenopausal women with obesity. Given the cognitive and health benefits of both dietary intervention and physical-cognitive exercise, we hypothesized that each intervention would be beneficial to postmenopausal women with obesity and the combined interventions may potentially provide additive or synergistic effects on cognitive function. To further extend the understanding of the cardiometabolic changes linking these interventions to cognition, this study also investigated the effects of diet, physical-cognitive exercise, and combined intervention on cardiometabolic outcomes of postmenopausal women with obesity. Identifying the most effective intervention to prevent or delay cognitive decline in postmenopausal women with obesity would have important clinical implications.

## Materials and methods

### Study design and participants

The study was a 3-month, assessor-blinded, four-arm randomized controlled trial. Permuted block randomization (random block sizes) was used to allocate all participants into four groups with a 1:1:1:1 ratio using a computer-generated random number sequence. Participants were eligible to participate if they were postmenopausal women aged between 45 and 59 years old with body mass index (BMI) ≥ 25 kg/m^2^ and waist-to-hip ratio (WHR) ≥ 0.80, had a sedentary lifestyle (exercise less than 1 h/week), had normal scores of Mental State Examination T10 (MSET10), and had a device that supports YouTube platform and application. Participants were considered ineligible if they had medical conditions that would limit physical exercise, had neurological conditions and depressive symptoms, had unstable body weight (weight variation greater than 5 kg in 3 months), had other dietary interventions (e.g., veganism, Mediterranean, and DASH diets), had an uncorrected hearing or visual impairment, and received hormone therapy. Written informed consent was obtained from all participants. The study protocol was approved by the Human Ethical Review Board of the primary investigator’s institution (AMSEC-63EX-102) and registered on ClinicalTrials.gov (identifier: NCT04768725). The study protocol of this trial has been published [25].

### Interventions

#### Dietary intervention

Participants in the dietary intervention group (diet group) and combined intervention group (combined group) received a dietary program. The dietary protocol was modified from previous studies [26, 27]. The gradually reduced calorie consumption was used to facilitate adherence to diet prescription and promote metabolic and behavioral adaptation to fasting. The participants consumed a self-selected dietary intake with 25–75% of their estimated energy requirements for 2 days/week (75% of energy requirements for weeks 1–4, 50% of energy requirements for weeks 5–8, and 25% of energy requirements for weeks 9–12) and consumed *ad libitum* on the remaining 5 days. As for energy needs, the estimated baseline energy requirements were performed using self-reported physical activity (METs) × estimated basal metabolic rate. All food details (food types, the amount of food in each meal, and portion sizes of food) were self-recorded by the participant via a mobile phone application. Total caloric intake (kcal/day) was estimated and calculated based on the nutritive values of Thai foods, Bureau of Nutrition [28] via a mobile phone application. Before beginning the intervention, all participants were trained by a researcher to ensure that they could calculate and record their dietary intake.

### Physical-cognitive exercise

Participants in the physical-cognitive exercise group (exercise group) and combined group participated in a home-based, physical-cognitive program for 60 min/day, 3 days/week for 3 months. The program consisted of 10 min for warm-up, 40 min for physical-cognitive exercise, and 10 min for cool down which was incorporated on YouTube. There were three main training sessions, which included the following: (1) physical session (moderate intensity of resistance and aerobic exercise), (2) simultaneous physical-cognitive session (challenging cognitive ability while moving upper and lower extremities), and (3) cognitive session. As for the physical part, the resistance exercise component involved major muscle groups of upper and lower extremities, using cuff weight and the participant’s body weight, while the aerobic exercise component involved aerobic dance exercise including high knee marching, cha cha cha, and two side steps. For the cognitive part, cognitive training that covered neurocognitive subdomains including attention, executive function, and memory (e.g., remembering animal pictures while moving, solving mathematical puzzle questions, listening to a short story and remembering the content of the story as much as possible while moving upper and lower extremities) were included in the program. For the progression of training, the speed of movement, the number of repetitions, the direction of movement, and the number of aerobic dance steps were used to grade the level of physical demand. Additionally, memory load, the number of stimuli, and the amount of attention were used to grade the level of cognitive demand. Prior to the home-based exercise, participants were asked to practice with a researcher and received a link to a YouTube video that consisted of instructional explanations of each session.

### Combined dietary intervention and physical-cognitive exercise

Participants in the dietary and physical-cognitive intervention group (combined group) followed the dietary and physical-cognitive exercise protocol. They were instructed to exercise on *ad libitum* days of the dietary intervention.

### Control condition

Participants in the control group were asked to continue daily routine activities and usual lifestyle behavior without any prescribed physical activity or diet.

### Outcome assessments

#### Primary outcome measures

Subdomains of executive function including the task-switching ability and inhibiting interference were assessed by the Trail Making Test (TMT) [29] and Stroop Color and Word Test (SCWT) [30], respectively. The difference in times to complete TMT part B and part A (TMT B-A) was used to reflect the switching ability between tasks [29]. For SCWT, the number of correct answers in the word (W), color (C), and color-word (CW) conditions were used to determine the interference score (IG) following the formula: IG = CW − [(W × C)/(W + C)] [30]. A lower IG score indicates a greater difficulty in inhibiting interference. In addition, episodic memory was assessed using a logical memory test [31]. The scores of delayed recall from two short stories were collected. A higher score indicates a greater performance of memory. For plasma brain-derived nerve growth factor (BDNF) levels, a commercially available quantitative sandwich enzyme-linked immunosorbent assay (ELISA) kit was used for BDNF measurement, following standard protocols provided by the test manufacturer (ab212166, Abcam).

#### Secondary outcome measures

Global and specific cognitive domains including attention and language domain were assessed by the Montreal Cognitive Assessment (MoCA) test [32], the Digit Span Test [33], and the verbal fluency test [34], respectively. A higher score on the MoCA test, Digit Span Test, and fluency test indicate better cognitive performance.

For metabolic parameters, plasma total cholesterol, glucose, and triglyceride levels were assessed using colorimetric assay kits, and insulin levels using a sandwich ELISA kit. In addition, insulin resistance was calculated using the homeostasis model assessment of insulin resistance (HOMA-IR) index following the formula: HOMA-IR index = fasting glucose (mg/dL) X fasting insulin (µU/mL) / 405. In addition, plasma adiponectin and interleukin (IL)-6 levels were determined using the adiponectin human ELISA kit (KHP0041, Thermo Fisher Scientific, MA) and the IL-6 human ELISA kit (BMS213HS, Thermo Fisher Scientific, MA), respectively.

Physical fitness was measured using 6-minute walk test (6MWT) [35], muscle strength was measured using hand grip strength test for the upper extremity strength [36], and 30-second Chair Stand Test (30-s CST) for the lower extremity strength [37]. The predicted maximal oxygen consumption (VO_2_ max) was calculated indirectly following the equation: VO_2_ max (ml.kg^− 1^.min^− 1^) = 26.9 + 0.014 × Meters walked in 6MWT– 0.38 x BMI (kg/m^2^) [38]. In addition, body weight and body composition were measured using a bioelectrical impedance analyzer (Tanita BC-418, Tokyo, Japan).

### Adherence and adverse events

To maximize the accuracy of the information, participants were instructed to record their exercise and/or their food intake in the logbook and/or the mobile application right after exercise or every fast day. A research team member made weekly contact with each participant to provide encouragement, facilitate adherence, and receive feedback. Additionally, participants were instructed to report diet- and exercise-related adverse events occurring during the study.

### Sample size calculation

The sample size in this study was determined for the primary outcomes based on previous findings [39–41]. The smallest effect size (0.38 for plasma BDNF [41]) was chosen. With a power of 80%, 5% type I error, and 15% dropout, 92 participants (23 participants per group) were required.

### Statistical analyses

Descriptive statistics were used to describe the demographic characteristics of the participants. The normality of the data was tested using the Shapiro-Wilk test. Demographic variables were compared using one-way ANOVA. Data were analyzed using the intention-to-treat (ITT) approach. The multiple imputations procedure was used for handling the missing data. ANCOVA with baseline values and education level (for cognitive outcomes) as covariates were conducted to compare the difference in outcome measures among groups. Bonferroni correction was used for post hoc pairwise comparisons. Partial eta squared values (η_p_^2^) were reported as measures of effect size (ES) [42]. SPSS software (version 25.0, IBM Corporation, Chicago, IL, USA) was used for all statistical analyses. A significant level was set at *p* < 0.05, two-sided.

## Results

**Fig. 1:**
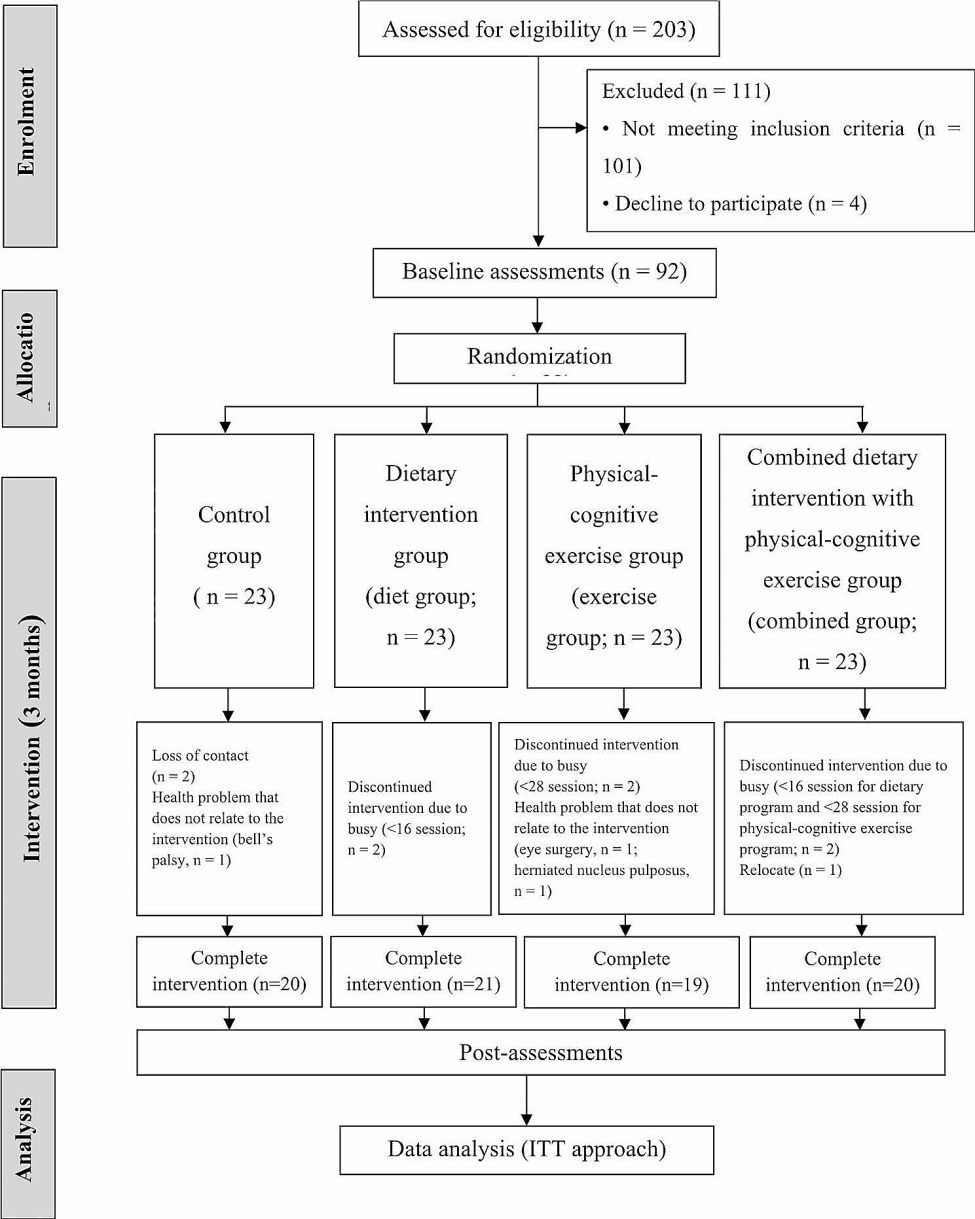
CONSORT diagram showing flow of patients through the study

Of 203 participants screened for eligibility, 92 were enrolled and randomly assigned to the diet, exercise, combined, or control group (23 per group). Twelve participants did not complete the study (3 in the control group, 2 in the diet group, 4 in the exercise group, and 3 in the combined group), resulting in a dropout rate of 13.04%. Mean intervention adherence over the 3 months was 88.83% for the diet group and 92.11% for the exercise group. As for the combined group, mean adherence was 87.30% for the dietary program and 93.01% for the exercise program. Adverse events were not reported by any participants. The Consolidated Standards of Reporting Trials (CONSORT) flow diagram of enrollment, allocation, intervention, and analysis is shown in Fig. 1.

### Baseline characteristics of all participants

The baseline characteristics of all study participants are presented in Table 1. There is no significant difference between groups in age, education, weight, BMI, WHR, types of medicine, MSET10 score, and HADS score (Table 1).

**Table 1:** Participants demographic characteristics

Characteristic	Control group, *n* = 23	Diet group, *n* = 23	Exercise group, *n* = 23	Combined group, *n* = 23	p-value
Age (years)	53.61 ± 2.81	52.87 ± 3.88	52.70 ± 3.60	52.17 ± 3.35	0.562
Education (years)	13.74 ± 4.74	13.57 ± 3.89	14.35 ± 3.19	13.96 ± 3.30	0.911
Weight (kg)	71.31 ± 11.49	69.43 ± 7.19	74.83 ± 11.18	71.28 ± 11.16	0.360
BMI (kg/m^2^)	29.18 ± 2.85	28.28 ± 2.78	29.06 ± 2.90	29.73 ± 4.57	0.538
WHR	0.90 ± 0.06	0.87 ± 0.04	0.88 ± 0.04	0.88 ± 0.05	0.185
Types of medicine (number)	0.70 ± 0.97	0.52 ± 0.67	0.65 ± 0.71	0.57 ± 0.73	0.869
MSET10 (score)	27.52 ± 1.27	27.17 ± 1.53	27.26 ± 1.36	27.65 ± 1.11	0.586
HADS (score)	3.43 ± 3.20	2.65 ± 1.64	3.52 ± 2.63	3.35 ± 2.46	0.642

All values are means ± standard deviations (SD). Types of medicine (antihypertensive, antidyslipidemic, or antidiabetic medication). BMI = body mass index, WHR = waist-to-hip ratio, MSET10 = Mental State Examination T10 (total score = 29 points), HADS = Hospital Anxiety and Depression Scale

### The comparative effect of diet, physical-cognitive exercise, and combined interventions on cognitive function

**Fig. 2:**
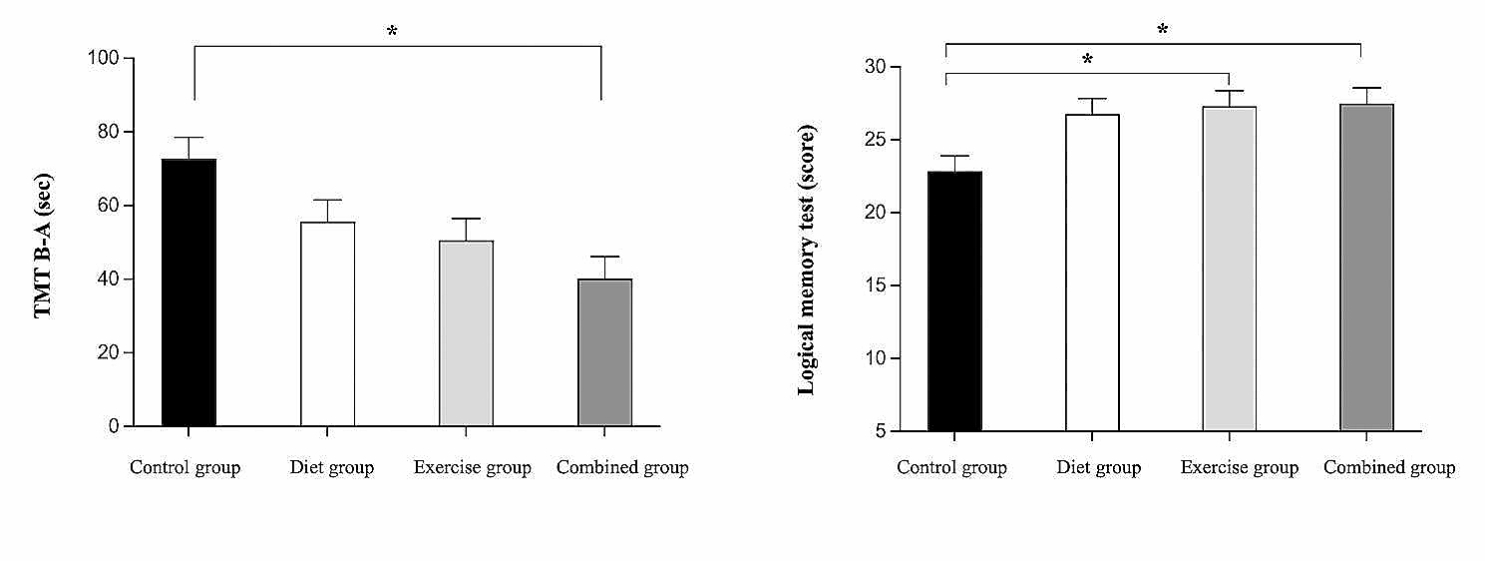
Cognitive test measures at 3-month retest for each group Post Hoc test indicated significant difference between groups at *p* < 0.05, scores are adjusted for education and baseline values. * = Significantly difference when compared to control group, TMT B-A = Trail Making Test part B-A

The results for cognitive function (baseline and the end of 3 months) are presented in Table 2. At the post-3-month intervention, results of the ANCOVA indicated significant group effects for TMT B-A and the logical memory test. Post hoc analyses found that the combined group demonstrated a significant reduction in TMT B-A score when compared to the control group (*p* < 0.05, Fig. 2A). In addition, the exercise and combined group demonstrated significantly higher scores in logical memory test when compared to the control group (*p* < 0.05, Fig. 2B). There was no significant difference among combined, diet, and exercise groups in any cognitive outcomes (*p* > 0.05).

**Table 2:** Mean score (SD) in cognitive test measures between baseline and 3-month retest for each group

Outcome variables	Control group, *n* = 23		Diet group, *n* = 23		Exercise group, *n* = 23		Combined group, *n* = 23		p-value ^a^	η_p_^2^
	Baseline	3-Month	Baseline	3-Month	Baseline	3-Month	Baseline	3-Month		
MoCA (score)	23.91 ± 3.64	25.01 ± 3.02	24.70 ± 3.66	26.10 ± 2.94	24.96 ± 2.36	25.95 ± 2.38	24.52 ± 2.29	25.85 ± 2.31	0.687	0.017
TMT B-A (sec)	58.65 ± 29.14	73.17 ± 41.64	60.28 ± 39.86	56.92 ± 26.83	53.52 ± 29.70	48.73 ± 29.08	58.41 ± 22.87	40.40 ± 20.74	0.002	0.156
Logical memory test (score)	21.09 ± 7.39	22.49 ± 7.18	22.26 ± 9.15	27.09 ± 8.98	21.87 ± 6.60	27.50 ± 4.24	21.48 ± 6.59	27.41 ± 6.19	0.007	0.131
SCWT (IG score)	−4.90 ± 6.60	−4.85 ± 6.73	−0.94 ± 5.19	1.28 ± 6.90	−2.71 ± 7.74	−0.61 ± 5.16	−2.68 ± 6.25	0.20 ± 6.59	0.053	0.085
Verbal fluency test (score)	20.96 ± 5.17	22.55 ± 5.18	20.96 ± 4.24	23.87 ± 5.29	22.70 ± 5.52	24.92 ± 4.65	21.57 ± 4.17	24.45 ± 4.89	0.539	0.025
Digit span test (score)	13.74 ± 3.48	13.80 ± 3.71	14.35 ± 2.76	14.52 ± 3.50	15.74 ± 2.94	15.88 ± 3.13	13.48 ± 3.30	14.99 ± 4.45	0.216	0.050

Note: High scores in the MoCA, Logical memory test, SCWT, Verbal fluency test, Digit span test, and low scores in TMT B-A indicate greater cognitive performances

MoCA = Montreal Cognitive Assessment test (0–30 points), TMT B-A = Trail Making Test part B-A, SCWT = Stroop Color and Word Test

^a^Analysis of covariance comparing group differences at 3 months adjusted for education and baseline values. All values are means ± standard deviations (SD)

### The comparative effect of diet, physical-cognitive exercise, and combined interventions on metabolic parameters and plasma biomarkers

**Fig. 3:**
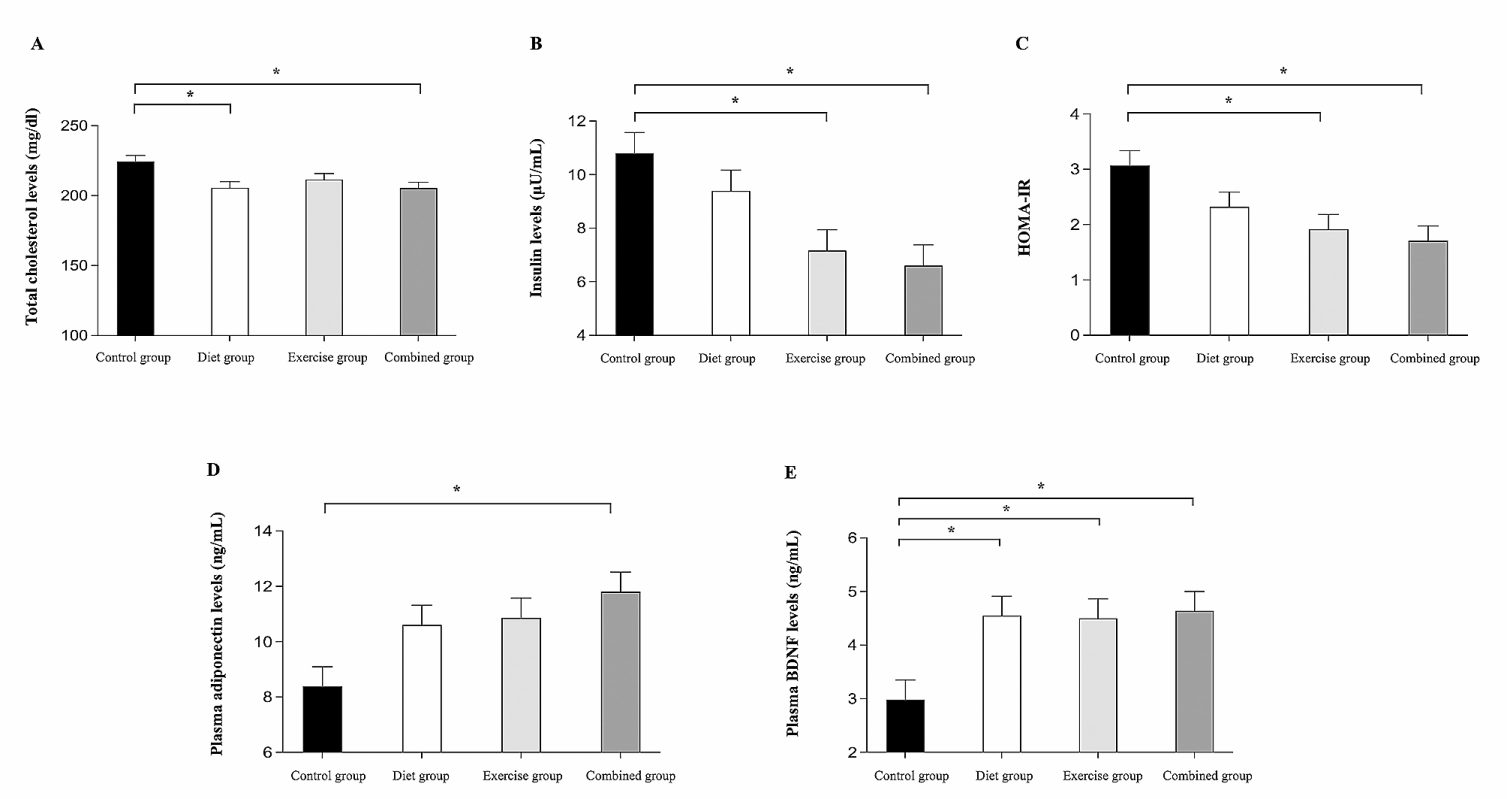
Metabolic parameters and associated plasma biomarkers at 3-month retest for each group. Post Hoc test indicated significant difference between groups at *p* < 0.05, scores are adjusted for baseline values. * = Significantly difference when compared to control group, HOMA-IR = The homeostasis model assessment of insulin resistance, BDNF = Brain-derived nerve growth factor

The results for metabolic parameters and plasma biomarkers (baseline and the end of 3 months) are shown in Table 3. There were significant group effects for plasma cholesterol levels, insulin levels, HOMA-IR index, adiponectin levels, and BDNF levels at the end of the 3-month intervention. Post hoc analyses revealed that both the diet and combined intervention groups showed a significant reduction in total cholesterol levels when compared with the control group (*p* < 0.05, Fig. 3A), while both the exercise and combined intervention groups showed a significant reduction in insulin levels and HOMA-IR index when compared with the control group (*p* < 0.05, Fig. 3B, C). Only the combined intervention group showed significant elevation in plasma adiponectin levels when compared with the control group (*p* < 0.05, Fig. 3D). All three intervention groups (diet, exercise, and combined groups) showed significant increment in plasma BDNF levels when compared with the control group (*p* < 0.05, Fig. 3E). Nevertheless, there was no significant difference in plasma cholesterol levels, plasma insulin levels, HOMA-IR index, plasma adiponectin levels, and plasma BDNF levels among combined, diet, and exercise groups (*p* > 0.05).

**Table 3:** Mean score (SD) in metabolic parameters and biomarker test measures between baseline and 3-month retest for each group

Outcome variables	Control group, *n* = 23		Diet group, *n* = 23		Exercise group, *n* = 23		Combined group, *n* = 23		p-value ^a^	η_p_^2^
	Baseline	3-Month	Baseline	3-Month	Baseline	3-Month	Baseline	3-Month		
Total cholesterol levels (mg/dl)	213.52 ± 42.91	221.57 ± 49.71	225.39 ± 46.56	213.88 ± 47.26	217.65 ± 33.91	212.42 ± 39.52	210.26 ± 41.36	199.41 ± 37.30	0.006	0.134
Triglyceride levels (mg/dl)	135.70 ± 62.88	147.91 ± 81.56	144.48 ± 82.72	130.10 ± 62.75	155.09 ± 80.59	132.59 ± 55.37	147.52 ± 78.29	129.20 ± 59.13	0.095	0.070
Glucose levels (mg/dl)	110.65 ± 38.94	110.55 ± 38.28	99.87 ± 10.61	97.63 ± 10.84	115.87 ± 42.84	115.11 ± 39.59	101.35 ± 11.39	101.29 ± 10.20	0.688	0.017
Insulin levels (µU/mL)	12.95 ± 5.95	10.75 ± 4.89	12.83 ± 7.43	9.33 ± 4.94	14.25 ± 9.42	7.33 ± 2.49	13.08 ± 4.47	6.57 ± 2.15	0.001	0.181
HOMA-IR	3.47 ± 1.76	3.08 ± 2.25	3.15 ± 1.81	2.23 ± 1.15	3.94 ± 2.67	2.06 ± 0.98	3.27 ± 1.20	1.66 ± 0.58	0.003	0.151
plasma IL-6 levels (pg/mL)	0.82 ± 0.82	1.08 ± 1.25	0.65 ± 0.36	0.77 ± 0.42	0.73 ± 0.40	0.77 ± 0.45	0.64 ± 0.33	0.68 ± 0.37	0.304	0.041
plasma adiponectin levels (ng/mL)	6.73 ± 2.68	8.45 ± 3.51	6.63 ± 1.40	10.63 ± 3.42	6.18 ± 1.72	10.71 ± 3.32	6.79 ± 1.58	11.89 ± 3.57	0.008	0.126
plasma BDNF levels (ng/mL)	3.36 ± 2.89	2.89 ± 1.72	3.21 ± 2.43	4.39 ± 1.80	4.01 ± 2.23	4.67 ± 2.15	3.79 ± 2.17	4.72 ± 2.25	0.004	0.142

^a^Analysis of covariance comparing group differences at 3 months adjusted for baseline values. All values are means ± standard deviations (SD). IL-6 = Interleukin 6, BDNF = Brain-derived nerve growth factor

### The comparative effect of diet, physical-cognitive exercise, and combined interventions on body weight and body composition

**Fig. 4:**
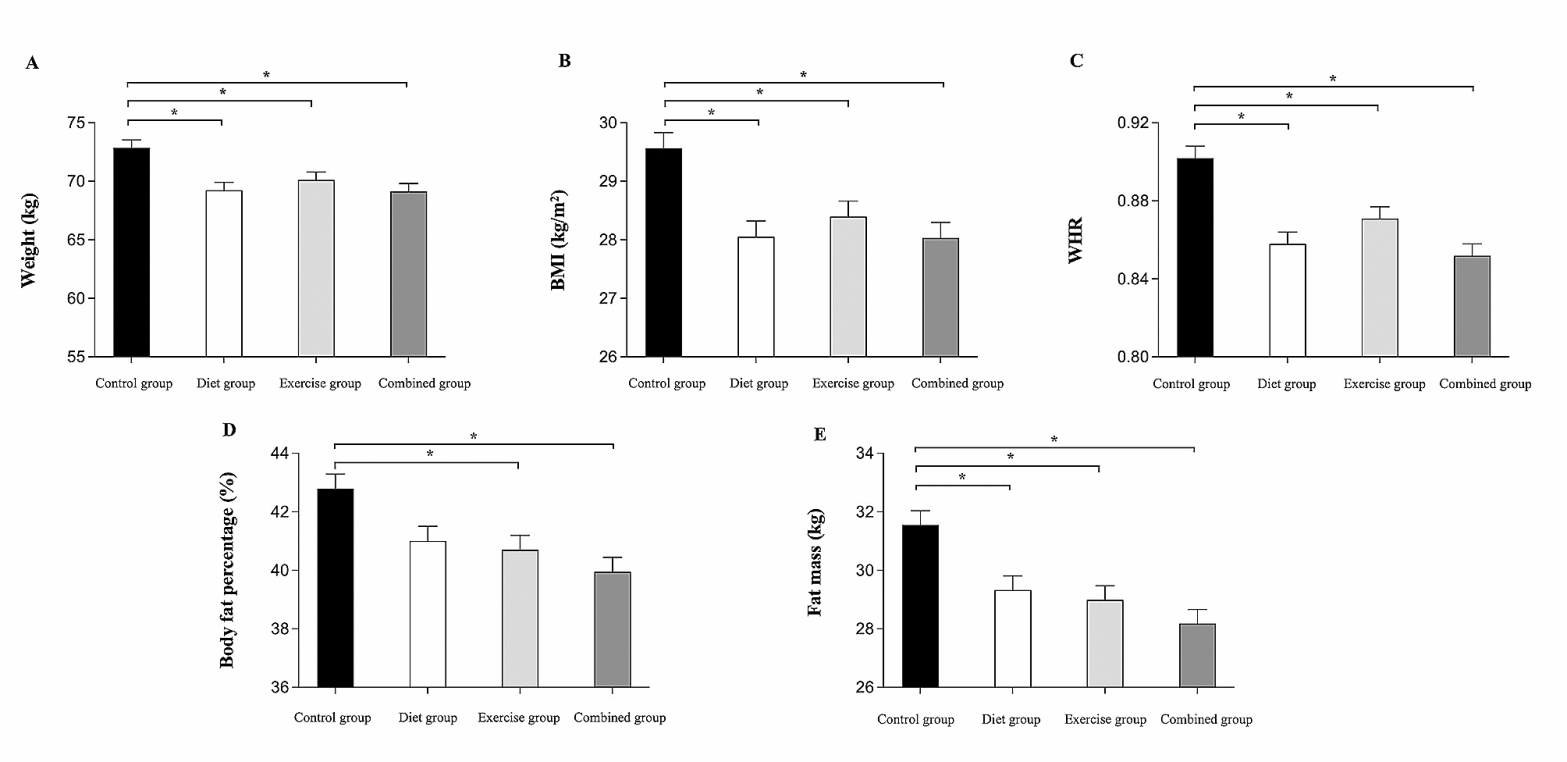
Body weight and body composition test measure at 3-month retest for each group. Post Hoc test indicated significant difference between groups at *p* < 0.05, scores are adjusted for baseline values. * = Significantly difference when compared to control group, BMI = Body mass index

The results for weight and body composition variables (baseline and the end of 3 months) are presented in Table 4. At the post-3-month intervention, results of the ANCOVA indicated significant group effects for weight, BMI, WHR, body fat percentage, and fat mass. Post hoc analyses found that participants in all three intervention groups demonstrated a significant reduction in weight, BMI, WHR, and fat mass (*p* < 0.05, Fig. 4A–C, E), whereas only participants in the exercise and combined intervention groups demonstrated a significant reduction in body fat percentage compared to the control group (*p* < 0.05, Fig. 4D). However, there was no significant difference in weight, BMI, WHR, body fat percentage, and fat mass among the three intervention groups (*p* > 0.05).

**Table 4:** Mean score (SD) in body weight and body composition test measures between baseline and 3-month retest for each group

Outcome variables	Control group, *n* = 23		Diet group, *n* = 23		Exercise group, *n* = 23		Combined group, *n* = 23		p-value ^a^	η_p_^2^
	Baseline	3-Month	Baseline	3-Month	Baseline	3-Month	Baseline	3-Month		
Weight (kg)	71.31 ± 11.49	72.49 ± 11.47	69.43 ± 7.19	67.04 ± 8.16	74.83 ± 11.18	73.12 ± 10.81	71.28 ± 11.16	68.72 ± 11.23	< 0.001	0.192
BMI (kg/m^2^)	29.18 ± 2.85	29.68 ± 2.86	28.28 ± 2.78	27.31 ± 3.16	29.06 ± 2.90	28.40 ± 2.76	29.73 ± 4.57	28.67 ± 4.67	< 0.001	0.206
WHR	0.90 ± 0.06	0.92 ± 0.06	0.87 ± 0.04	0.85 ± 0.05	0.88 ± 0.04	0.87 ± 0.04	0.88 ± 0.05	0.85 ± 0.05	< 0.001	0.302
Body fat percentage (%)	41.70 ± 4.21	42.19 ± 4.45	41.47 ± 3.81	40.18 ± 4.15	42.81 ± 3.41	41.21 ± 4.33	43.22 ± 5.69	40.87 ± 6.49	0.001	0.167
Fat mass (kg)	30.09 ± 7.91	30.95 ± 8.12	29.00 ± 5.37	27.61 ± 5.79	32.33 ± 7.43	30.65 ± 8.21	31.36 ± 8.98	28.86 ± 9.33	< 0.001	0.239
Fat free mass (kg)	41.22 ± 4.20	41.54 ± 4.17	40.44 ± 2.72	40.47 ± 2.79	42.51 ± 4.36	42.55 ± 3.97	39.93 ± 3.01	40.13 ± 3.18	0.846	0.009

^a^Analysis of covariance comparing group differences at 3 months adjusted for baseline values. All values are means ± standard deviations (SD). BMI = Body Mass Index, WHR = Waist-to-hip ratio

### The comparative effect of diet, physical-cognitive exercise, and combined interventions on physical function

**Fig. 5:**
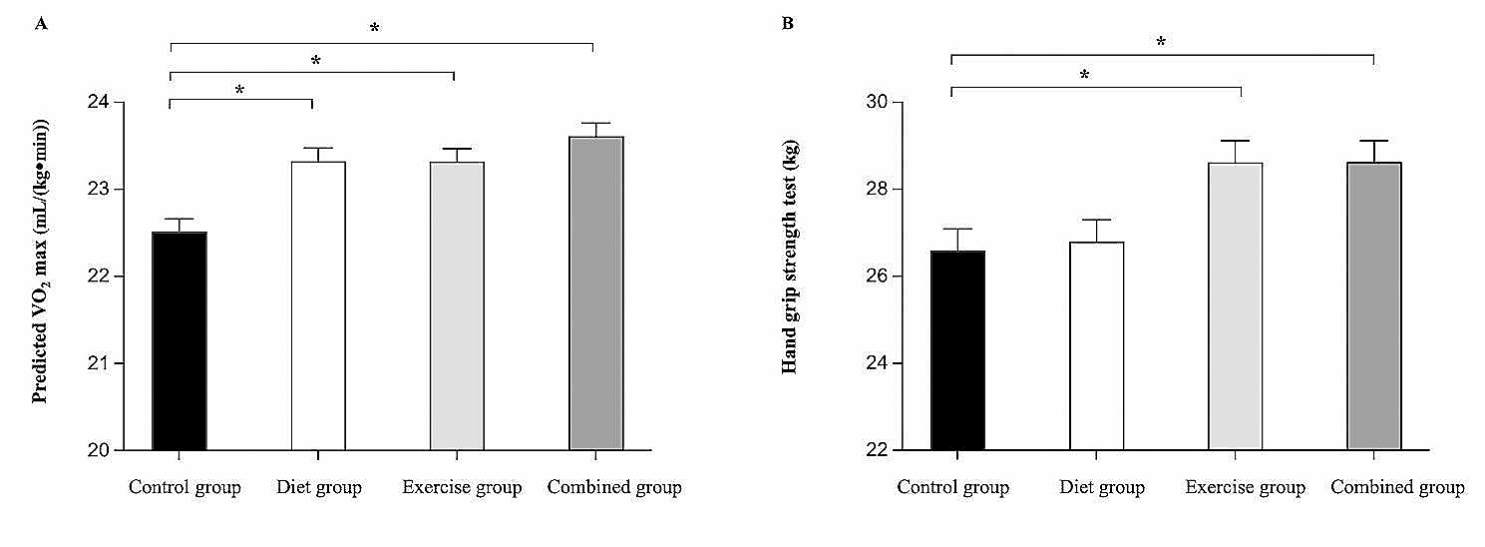
Physical test measure at 3-month retest for each group. Post Hoc test indicated significant difference between groups at *p* < 0.05, scores are adjusted for baseline values. * = Significantly difference when compared to control group, predicted VO_2_ max = Predicted maximal oxygen consumption

The results for cardiorespiratory fitness and muscle strength (baseline and at the end of 3 months) are shown in Table 5. Significant group effects for predicted maximal oxygen consumption and hand grip strength test were observed at the end of the 3-month intervention. Post hoc analyses demonstrated that while the three intervention groups showed significant improvement in predicted maximal oxygen consumption when compared with the control group (*p* < 0.05, Fig. 5A). Only the exercise and combined intervention groups showed greater improvements in hand grip strength when compared with the control group (*p* < 0.05, Fig. 5B). Nevertheless, there was no significant difference in predicted maximal oxygen consumption and grip strength among combined, diet, and exercise groups (*p* > 0.05).

**Table 5:** Mean score (SD) in physical test measures between baseline and 3-month retest for each group

Outcome variables	Control group, *n* = 23		Diet group, *n* = 23		Exercise group, *n* = 23		Combined group, *n* = 23		p-value ^a^	η_p_^2^
	Baseline	3-Month	Baseline	3-Month	Baseline	3-Month	Baseline	3-Month		
Predicted maximal oxygen consumption (VO_2_max) (mL/(kg•min))	22.31 ± 1.48	22.09 ± 1.58	23.18 ± 1.49	23.77 ± 1.68	22.99 ± 1.43	23.57 ± 1.49	22.46 ± 2.42	23.34 ± 2.54	< 0.001	0.258
Hand grip strength test (kg)	26.78 ± 5.96	26.56 ± 5.29	27.26 ± 4.59	27.13 ± 3.28	26.54 ± 4.66	28.40 ± 4.54	26.74 ± 4.38	28.56 ± 4.57	0.003	0.150
30-s chair stand test (time)	14.57 ± 4.71	15.05 ± 4.48	14.91 ± 4.68	17.03 ± 5.09	15.09 ± 2.37	17.39 ± 2.82	14.87 ± 3.43	16.98 ± 3.96	0.134	0.062

^a^Analysis of covariance comparing group differences at 3 months adjusted for baseline values. All values are means ± standard deviations (SD). VO_2_max = Maximal oxygen consumption

## Discussion

To the best of our knowledge, the present study is the first randomized controlled trial study to directly compare the independent and combined effects of dietary intervention and physical-cognitive exercise on cognitive function and cardiometabolic health in postmenopausal women with obesity. Findings from our study showed that after 3 months of intervention, cognitive function was unchanged in the dietary intervention group, whereas memory improvement was shown in the exercise and combined intervention groups when compared to the control group. In line with cognitive findings, improvements in insulin sensitivity and plasma BDNF levels were identified in the exercise and combined intervention groups. It is worth noting that improvement in executive function which was accompanied by elevation of plasma adiponectin levels was only demonstrated in the combined intervention group.

Promoting brain health earlier in life can contribute to a lower risk of developing cognitive impairment in later life [43]. Our findings demonstrated that memory improvement was illustrated in the exercise and combined interventions, but not dietary intervention, suggesting that physical-cognitive exercise is likely to be the driving force for memory improvement. The exercise program in the present study consisted of physical-cognitive exercise that targeted improvement in both physical health and specific cognitive domain including memory. Research evidence has demonstrated that physical-cognitive exercise positively impacts on memory-related outcomes [24, 44]. Although the mechanisms underlying the effects of physical-cognitive exercise on memory improvement have not been well understood, both animal and human studies have proposed that the alteration in supramolecular levels (e.g., mitochondrial biogenesis, neurotrophic factors, and insulin sensitivity) and structural/functional changes in the hippocampus after exercise or cognitive training, could lead to memory improvement [24, 45–47]. The present findings are in agreement with previous studies that found significant increases in plasma BDNF level [24] or insulin sensitivity [22, 23] along with memory improvement after physical-cognitive training. Together, these findings suggest that plasma BDNF level and insulin sensitivity might be one mechanism responsible for memory improvement following physical-cognitive exercise. The present study is one of the first to reveal this phenomenon in postmenopausal women with obesity and further suggests that physical-cognitive exercise either with or without dietary intervention was effective in improving memory.

Among the three interventional groups, only the combined intervention improved executive function as assessed by the TMT B-A. To the best of our knowledge, only one previously published study by Napoli et al. [39] investigated the independent and combined effects of dietary intervention (continuous calorie restriction) and exercise (multicomponent training) on cognitive function. They found that while neither dietary intervention nor exercise alone established the improvement in executive function, combined intervention improved executive function (measured by TMT) as compared to controls in older adults with obesity [39]. Additionally, another study by Peven et al. [48] reported that combined dietary intervention (continuous calorie restriction) with high-intensity exercise improved executive function in adults with overweight and obesity, without improvement in executive function in dietary intervention alone. Altogether, findings from previous studies and our study that improvement in executive function was demonstrated in the combined interventions but not in single dietary or exercise intervention suggest that combining both interventions might exert an additive effect on executive function.

To better understand the changes in cardiometabolic health linking the effects of diet, physical-cognitive exercise, and combined intervention to cognitive function, associated plasma biomarkers were determined in our study. The present study revealed that only the combined intervention improved plasma adiponectin levels in postmenopausal women with obesity. A systematic review and meta-analysis study demonstrated that combined dietary intervention with exercise is an effective method for increasing the concentration of adiponectin in individuals who are overweight or obese [49]. Peripheral adiponectin can cross the blood-brain barrier and act directly in the brain by modulating and signaling through adiponectin receptors which express in the cortex, hippocampus, and hypothalamus to provide neuroprotective effects [50]. Although the link between adiponectin and cognitive function, especially in the executive domain has not been well understood, a positive association between executive function and adiponectin levels in postmenopausal women with obesity was reported in a previous study [3]. To further explore this issue, an additional analysis was conducted to determine the association between change in executive function and plasma adiponectin levels from baseline to post-3-month intervention. Consistent with previous findings, Pearson’s correlation coefficient revealed that there is a positive association between executive function and plasma adiponectin levels (*p* = 0.047, *r* = 0.207). Given the multiple roles of adiponectin including metabolism control, regulation of insulin sensitivity, and stimulation of neural plasticity [50], it is possible that adiponectin may potentially be a key mediator linking the effects of combined intervention and improvement in executive function of postmenopausal women with obesity.

Apart from adiponectin, insulin sensitivity might also play a critical role in executive function. Schuur et al. [51] reported that insulin resistance is associated with a decline in executive function in women. In the present study, the HOMA-IR index in the combined intervention group changed from having insulin resistance at baseline (3.27) to normal levels (1.66) after 3 months of intervention. Therefore, the improvement in insulin sensitivity could be another potential mediator linking the effects of combined intervention and improvement in executive function in postmenopausal women with obesity.

Dietary intervention with IF has been recommended as one of several dietary intervention strategies for achieving weight loss and preventing obese-related comorbidity in individuals with overweight and obese conditions [52, 53]. The present findings that dietary intervention with IF for 3 months reduced body weight, decreased BMI, preserved fat-free mass, and attenuated cholesterol levels support previous recommendations. To date, studies investigating the effects of IF on cognitive function are limited and findings are inconclusive. For example, Kim et al. [27] reported that IF (2 days/weeks, 4 weeks) increased pattern separation, but not recognition memory in adults with obesity. Recently, a 3-year longitudinal study by Ooi et al. [53] demonstrated that regularly IF practice (2 days/weeks, 3 years) improved global cognition, attention, and memory of older adults. IF duration might partly influence cognitive changes. Further, although our study demonstrated improved weight outcomes and plasma BDNF levels, insulin sensitivity did not improve following IF. Ooi et al. [53] suggested that insulin sensitivity may be the key mediator for cognitive improvement following IF intervention. Taken together, it is posited that the relatively short duration of IF and the lack of improvement in insulin sensitivity might account for the unchanged cognitive performance of postmenopausal women with obesity in the present study. Further clinical trials with longer IF duration should be conducted to confirm this postulation.

As for physical function, several studies have documented that either dietary intervention or physical-cognitive exercise could improve physical health and reduce cardiovascular risks [10, 19]. Results from our study revealed that dietary intervention, physical-cognitive exercise, and combined intervention for 3 months increased weight loss and enhanced cardiorespiratory fitness in postmenopausal women with obesity, suggesting that all interventions are effective for weight management and promoting fitness. In addition to physical fitness, muscle strength is another important aspect of physical status. A previous study reported that grip strength is associated with the strength of the upper limb function and the general strength of the body [54]. A systematic review and meta-analysis study demonstrated the positive influence of physical-cognitive training on muscle strength including grip strength [55]. In accordance with the literature, participants who engaged in physical-cognitive exercise in the present study demonstrated a significant gain in grip strength. Nevertheless, strength gain was not observed for the lower extremity muscles as measured by the 30s-CST. It is possible that the 30s-CST, which is commonly used to assess lower extremity strength in older adults [56], might not be sensitive enough to detect changes in lower limb muscle strength in middle-aged obese women. Further studies with more sensitive measurements (e.g., isokinetic dynamometer) are warranted to confirm the study findings.

The study hypothesis was partially supported in that the combined intervention improved a wider range of cognitive subdomains (memory and executive function) than the single intervention. However, such improvement was not significantly greater than either a single exercise or dietary intervention. In addition, some secondary outcomes (global cognitive function, attention, inhibiting interference, language domain) were unchanged after the intervention. We postulated that the dose (intensity and duration) of the intervention might be suboptimal for participants who had both obesity and postmenopausal conditions. While we tried to maximize safety and adherence, the intensity and duration of the intervention in the present study may have been insufficient to exert the synergistic effects and unveil changes in some cognitive outcomes. Furthermore, effect sizes, as reflected by the partial eta-squared, of most cognitive outcomes (Table 2) indicated that the interventions had small effects. Thus, a larger sample size might be required to detect significant changes in several outcome measures.

During the intervention period, the research team delivered weekly conceived and planned instructions to promote participant engagement and ensure that they could follow the intervention protocol. It should be noted that findings in the present study were due not only to the intervention program itself but also to the intervention fidelity delivered by the research staff. It is uncertain whether the findings would have been the same without the contribution of these strategies. Thus, the external validity of the present study findings should be interpreted with caution. To implement and generalize research findings, both the intervention program and fidelity should be considered.

The main strength of this RCT study is the inclusion of 4 arms (3 intervention arms and control), which allowed systematic comparisons of single and combined dietary and exercise interventions to each other and control condition. There are several limitations in this study that need to be acknowledged. First, unsupervised, self-recorded dietary intake and exercise adherence may be subject to inaccuracies. Nevertheless, given the nature of a home-based program, this limitation is inevitable. Second, the diagnosis of menopause in the present study was clinical-based. While it is a standard method for women aged over 45 years, the evaluation of estrogen levels to confirm postmenopausal conditions was not evaluated. Third, a long-term follow-up was not determined in this study. Thus, it is unknown whether the cognitive improvement in the exercise and the combined group would be maintained over time, and it is unknown whether the stable cognitive function in the diet group would be changed afterward. Lastly, as the sample size was not calculated for the secondary outcomes, the study may have been underpowered, unable to reveal the effects of the interventions on those outcomes.

## Conclusion

The combined intervention appears to improve a broader range of cognitive performance than single intervention. As the summation of health benefits in single dietary and exercise intervention was observed in the combined intervention, our results suggest that the combination of physical-cognitive exercise and dietary intervention for 3 months promotes additive effects on cognitive and health benefits of postmenopausal women with obesity. Adiponectin and insulin sensitivity could potentially serve as key mediators linking cognitive improvement in the combined intervention. Combined physical-cognitive exercise and dietary intervention is promising in improving the cognitive and physical health of postmenopausal women with obesity. However, further randomized controlled trial studies with an optimal intervention dose, long-term follow-up, and larger sample sizes are warranted to confirm the present findings.

### Electronic supplementary material

Below is the link to the electronic supplementary material.

**Additional file 1:** CONSORT checklist

**Additional file 2:** TIDieR checklist

## Data Availability

The datasets used and/or analysed during the current study are available from the corresponding author on reasonable request.

## Abbreviations

IF: Intermittent fasting
BMI: body mass index
WHR: waist-to-hip ratio
MSET10: Mental State Examination T10
TMT: Trail Making Test
W: word
C: color
CW: color-word
IG score: interference score
SCWT: Stroop Color and Word Test
ELISA: enzyme-linked immunosorbent assay
BDNF: brain-derived nerve growth factor
MoCA test: the Montreal Cognitive Assessment test
HOMA-IR index: the homeostasis model assessment of insulin resistance index
IL-6: interleukin-6
6MWT: 6-minute walk test
30-s CST: 30-second Chair Stand Test
VO_2_ max: maximal oxygen consumption
ITT: intention-to-treat
HADS: Hospital Anxiety and Depression Scale
